# Real-World Treatment Pathways of Adult Patients with Glioblastoma and Other CNS Tumors: A Population-Based Registry Study

**DOI:** 10.3390/curroncol33040236

**Published:** 2026-04-21

**Authors:** Eliana Ferroni, Alessandra Andreotti, Stefano Guzzinati, Susanna Baracco, Maddalena Baracco, Emanuela Bovo, Eva Carpin, Antonella Dal Cin, Alessandra Greco, Anna Rita Fiore, Laura Memo, Daniele Monetti, Silvia Rizzato, Jessica Elisabeth Stocco, Carmen Stocco, Sara Zamberlan, Marta Maccari, Alberto Bosio, Luca Denaro, Giampietro Pinna, Sara Lonardi, Giuseppe Lombardi, Manuel Zorzi

**Affiliations:** 1 Veneto Cancer Registry, Epidemiological Department, Azienda Zero, 35132 Padua, Italy; eliana.ferroni@azero.veneto.it (E.F.); stefano.guzzinati@azero.veneto.it (S.G.); susanna.baracco@azero.veneto.it (S.B.); maddalena.baracco@azero.veneto.it (M.B.); eva.carpin@azero.veneto.it (E.C.); antonella.dalcin@azero.veneto.it (A.D.C.); alessandra.greco@azero.veneto.it (A.G.); annarita.fiore@azero.veneto.it (A.R.F.); laura.memo@azero.veneto.it (L.M.); daniele.monetti@azero.veneto.it (D.M.); silvia.rizzato@azero.veneto.it (S.R.); jessicaelisabeth.stocco@azero.veneto.it (J.E.S.); carmen.stocco@azero.veneto.it (C.S.); sara.zamberlan@azero.veneto.it (S.Z.); manuel.zorzi@azero.veneto.it (M.Z.); 2 Department of Oncology, Oncology 1, Veneto Institute of Oncology IOV-IRCCS, 35128 Padua, Italy; marta.maccari@iov.veneto.it (M.M.); alberto.bosio@iov.veneto.it (A.B.); sara.lonardi@iov.veneto.it (S.L.); giuseppe.lombardi@iov.veneto.it (G.L.); 3 Department of Surgery, Oncology and Gastroenterology, University of Padua, 35128 Padua, Italy; 4 Academic Neurosurgery, Department of Neurosciences, University of Padua, 35128 Padua, Italy; luca.denaro@unipd.it; 5 Department of Neurosurgery, AOUI Borgo Trento Hospital, 37124 Verona, Italy; giampietro.pinna@aovr.veneto.it

**Keywords:** glioblastoma, central nervous system tumors, follow-up, tumor grade, cancer registry

## Abstract

**Background:** Population-level evidence on delivery of neuro-oncology care is essential for evaluating access, equity, and quality of treatment pathways. However, real-world data describing how patients with central nervous system (CNS) tumors, especially with glioblastoma, are managed across healthcare systems remain limited. This study aimed to characterize treatment pathways using linked registry and administrative data within a regional care network. **Methods:** All adult CNS tumors diagnosed between 2016 and 2020 were identified in the Veneto Cancer Registry. Tumor grading was derived using a validated text-mining algorithm, and surgical, radiotherapy, and systemic treatments were captured through linkage with regional healthcare utilization databases. Patterns of care were evaluated by tumor subtype, grade, and diagnostic pathway. **Results:** Among 1634 histologically confirmed tumors, glioblastoma represented the largest group. Surgical intervention was widely implemented, with high resection rates in glioblastoma and meningioma. Combined chemoradiotherapy constituted the primary adjuvant approach for glioblastoma and high-grade diffuse gliomas, whereas management of lower-grade tumors showed greater variability. Approximately one-third of patients received no oncologic therapy, primarily associated with older age or diagnostic uncertainty. Analysis of recurrent glioblastoma showed heterogeneous systemic treatment use, reflecting evolving therapeutic practice. **Conclusions:** Linking population-based registry and administrative data provides actionable insight into real-world delivery of neuro-oncology care, in particular for glioblastoma patients. This approach enables monitoring of treatment variability, identification of potential access gaps, and evaluation of system-level performance, supporting data-driven planning of multidisciplinary services and future quality improvement initiatives.

## Simple Summary

Population-based evidence on adult central nervous system (CNS) tumor management is scarce, particularly in Europe. Our study describes surgical and oncologic treatment pathways in patients with Glioblastoma and other CNS tumors resident in the Veneto Region (Northeastern Italy), by linking administrative healthcare data with the Cancer Registry data. Compared with prior studies, the authors report treatments by tumor types and grade, highlighting significant variation in treatment according to these tumor characteristics. By mapping complete patient pathways, the study helps to identify possible gaps in guideline adherence in clinical practice, providing information for quality improvement and supporting health policy planning.

## 1. Introduction

Primary brain and central nervous system (CNS) tumors affect both children and adults and can arise in all anatomical regions of the central nervous system. The vast majority (over 90%) occur in the brain, with the remaining cases affecting the meninges, spinal cord, and cranial nerves [1].

In adults, the most common histological type of primary CNS tumors is glioma, which encompasses a heterogeneous group of tumors. These range from high-grade gliomas, such as glioblastomas, to low-grade gliomas like astrocytomas and oligodendrogliomas. Other less common histological types include glial-origin tumors such as ependymomas and schwannomas, as well as medulloblastomas, CNS lymphomas, and meningiomas. According to the latest GLOBOCAN estimates produced by the International Agency for Research on Cancer (IARC), approximately 340,000 new cases of CNS tumors are diagnosed globally in 2025 [1].

Although CNS tumors account for only about 2% of all cancers, they represent a major source of morbidity and mortality worldwide. Primary brain tumors commonly manifest clinically at an advanced stage, when tumor growth or invasion of eloquent areas limits surgical resectability, with a negative impact on the 5-year overall survival rate, estimated at less than 35% for malignant CNS tumors [2].

In the 27-member European Union (EU27), approximately 28,000 CNS tumors are diagnosed annually [3]. Unlike some industrialized countries, such as the United States and the United Kingdom, where incidence rates have shown a steady increase, the incidence of CNS tumors in Italy has remained relatively stable in recent years [4,5]. However, while incidence and survival have been well characterized in recent years, data on treatment patterns and care pathways are still lacking.

Several key principles guide treatment options (surgery, systemic therapy, radiotherapy) for adults with brain tumors. Regardless of tumor histology, the best outcomes are generally achieved when neurosurgeons perform a maximal safe resection, removing as much of the tumor as possible while minimizing surgical morbidity and preserving neurological function. Equally important is obtaining an accurate diagnosis by collecting a sufficient amount of representative tumor tissue [6].

To our knowledge, data on the treatment of CNS tumors by histologic grade or differentiation are limited. The aim of our study was to describe the treatment approaches of CNS tumors, with a specific focus on the most common histological subtypes, especially glioblastoma.

## 2. Materials and Methods

The study cohort is represented by patients affected by glioblastoma and other CNS tumors, resident in the Veneto Region (almost 4.9 million inhabitants). We included all adult patients with a diagnosis of glioblastoma or other CNS tumor registered in the Veneto Cancer Registry (VCR) in the period 2016–2020.

The World Health Organization (WHO) 2016 classification [7], in use during the study period (2016–2020), was applied in order to map all CNS tumor subtypes. To ensure epidemiological relevance, the analysis was restricted to glioblastoma and other common CNS tumor types in the study population. Grade 2 and 3 meningiomas, considered clinically malignant, were also included in the cohort.

The CNS tumor groups analyzed were: glioblastoma, IDH-wildtype and IDH-mutant; astrocytoma grade 2–3; meningioma grade 2–3; oligodendroglioma grade 2–3; ependymoma grade 2–3; CNS embryonal tumor (medulloblastoma). This classification system enabled stratification of the cohort according to tumor biology and prognostic features, thereby providing the framework for subsequent analyses of treatment trajectories.

In order to analyze the clinical pathways of the study population, patients were individually linked to the main regional administrative healthcare databases. These include Hospital Admissions (HA), Outpatient Services (OPS), Drug Prescriptions (DP), and Hospital Drugs (HD). Surgical procedures for CNS tumors were identified within the HA database looking for specific ICD-9-CM (International Classification of Diseases, 9th Revision, Clinical Modification) codes [8], both in the primary procedure and in the secondary ones (01.11, 01.13 & 01.18, 01.14, 01.19, 01.23, 01.24, 01.25, 01.31, 01.39, 01.51, 01.52, 01.53, 01.59, 02.99, 03.09, 03.4). Within the HA database, systemic therapy (diagnosis codes V58.11–V58.12 or surgical procedure code 99.25) and radiotherapy (diagnosis code V58.0 or procedure codes 92.20–92.29) were also detected.

Within the OPS database, systemic therapy was traced using ICD-9-CM code 99.25.1, 99.25.2, while radiotherapy was identified using ICD-9-CM codes 92.20–92.29.

The pharmaceutical data flows, which comprise both HD and DP databases, were also used to identify systemic therapy treatments (ATC—Anatomical Therapeutic Chemical—codes L01AX03, L01EX05, L01AD02, L01AD05). An exploratory analysis was undertaken to characterize the subgroup of 918 patients with CNS tumors diagnosed through radiological imaging, without histopathological confirmation.

The full list of codes used for data extraction is provided in the Supplementary Materials.

In this study, the term “systemic therapy” was used to include both conventional chemotherapy and targeted agents (e.g., regorafenib; Bayer AG, Leverkusen, Germany), in order to more accurately reflect contemporary treatment strategies in neuro-oncology.

Descriptive statistical analyses were conducted considering clinical and demographic variables. Data were stratified by sex, age group (18–49, 50–69, and ≥70 years), morphology, and grade. For continuous variables, measures of central tendency and variability were reported as appropriate, while categorical variables were summarized using absolute and relative frequencies.

Statistical analyses were performed using R software (version 4.3.2; R Foundation for Statistical Computing, Vienna, Austria) [9], and SAS Enterprise Guide 8.3 software (SAS Institute Inc., Cary, NC, USA) [10].

## 3. Results

We described the follow-up of 1634 incident adult cases, diagnosed with glioblastoma and other CNS tumors from 2016 to 2020, and recorded in the Veneto Cancer Registry (VCR). This cohort included different histological subtypes of CNS tumors with intermediate to high-grade confirmed pathology.

### 3.1. Main Characteristics of Glioblastoma and Other CNS Tumors

Glioblastoma was the most common type of CNS tumor, accounting for 64.6% of cases, followed by meningiomas grade 2–3 (18.2%) and astrocytomas grade 2–3 (9.4%) (Table 1). Glioblastomas and astrocytomas were more frequently observed in males (61.4% and 57.5%, respectively), whereas meningiomas were more prevalent in females (58%).

Most CNS tumors were diagnosed in patients aged 50–69 years (50.2%), followed by those aged 70 and older (31.0%) and 18–49 years (18.8%). Glioblastomas were most common in the 50–69 age group (56.5%), while astrocytomas grade 2–3 and oligodendrogliomas grade 2–3 showed a younger age distribution, with 45.8% and 51.4% of cases occurring in patients aged 18–49, respectively (Table 1). High-grade meningiomas were more frequent in older patients (84.2% aged ≥50), while medulloblastomas predominantly affected the youngest group (73.3% aged 18–49).

**Figure 1:**
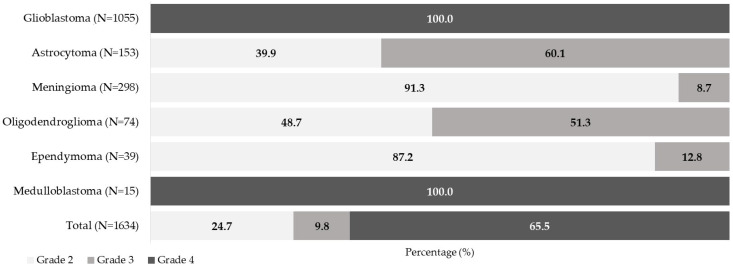
Distribution of tumor grade across CNS tumor subtypes (percent within subtype).

High-grade tumors (grade 4) accounted for the majority of cases (65.5%), with glioblastomas accounting for almost all of the grade 4 tumors in the cohort (98.6%) (Figure 1). Astrocytomas grade 2–3 and oligodendrogliomas grade 2–3 were more evenly distributed across grades 2 and 3, with 60.1% of astrocytomas and 51.3% of oligodendrogliomas classified as grade 3. Meningiomas and ependymomas were predominantly grade 2 (91.3% and 87.2%, respectively).

### 3.2. Patterns of Neurosurgical Management of Glioblastoma and Other CNS Tumors

**Figure 2:**
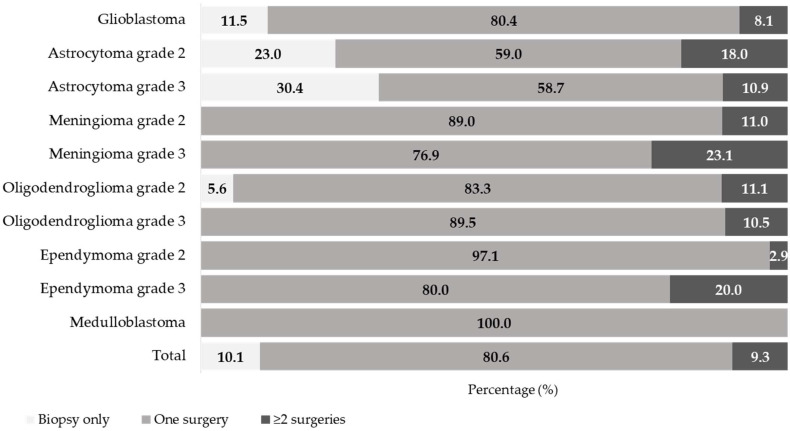
Neurosurgical management by tumor subtype (percent within subtype).

Most patients underwent one surgical intervention (80.6%), while 9.3% had two or more surgeries (due to recurrences or progression of disease) and 10.1% only had a biopsy (Figure 2).

Patients with glioblastoma were most likely to have one surgery (80.4%), whereas 11.5% just had a biopsy. Nearly one-third (23% and 30.4% for grades 2 and 3, respectively) of patients with astrocytomas only had a biopsy, whereas about 59% had a single surgical procedure. On the other hand, nearly all meningioma patients (89% grade 2 and 76.9% grade 3) had only one surgery. Similar trends have been observed for oligodendroglioma, ependymoma, and medulloblastoma, where the most frequent treatment was surgical resection (Figure 2).

The overall proportion of patients undergoing any neurosurgical procedure (biopsy or resection) remained stable between 2016 and 2020, indicating consistent access to surgical treatment across the study period. However, the proportion of patients undergoing more than one surgical procedure during the disease course increased, particularly among those with glioblastoma. In this group, repeated surgeries rose from approximately 8% in 2016 to more than 30% in 2020, suggesting a gradual shift toward a more proactive surgical approach in selected recurrent cases (Supplementary Materials Tables S1 and S2).

### 3.3. Patterns of Oncologic Treatments of Glioblastoma and Other CNS Tumors

Significant differences in the use of systemic therapy and radiotherapy among CNS tumor types and grades were found (Table 2).

Overall, 46.6% of patients underwent both systemic therapy and radiotherapy, 12.9% only systemic therapy, 8.6% only radiotherapy. One third of the study cohort (*n* = 521) received no therapy, of which 231 patients had meningioma grade 2 and 196 had glioblastoma. Astrocytoma grade 3 and glioblastoma patients received both treatments in 65.2% and 60.1% of cases, respectively. Grade 3 oligodendroglioma was frequently treated with both systemic therapy and radiotherapy (57.9%). Among patients with recurrent histologically diagnosed glioblastoma who received active therapy after progression, the most frequently used treatments were fotemustine (Servier, Suresnes, France) or lomustine (Bristol-Myers Squibb, New York, NY, USA) in approximately 70% of cases, whereas the remaining patients were treated with regorafenib, bevacizumab (Genentech Inc., South San Francisco, CA, USA), or temozolomide (Merck Sharp & Dohme B.V., Haarlem, The Netherlands) rechallenge.

Among low-grade gliomas (grade 2), postoperative oncologic treatment was frequently adopted. Specifically, 68.9% of astrocytoma grade 2 cases and 63.9% of oligodendroglioma grade 2 cases received adjuvant therapy with radiotherapy, systemic therapy, or both, whereas the remaining patients were managed with surgery alone (Figure 2). Among low-grade ependymomas, 85.3% of patients did not receive any oncological treatment.

Treatment patterns varied significantly by grade for meningiomas. Although most grade 2 meningiomas (84.9%) did not receive treatment, grade 3 patients were more likely to receive only radiotherapy (42.3%).

### 3.4. Summary Description of Surgical and Treatment Pathway of Patients with Glioblastoma and Other CNS Tumors

In general, 68.1% of patients had combined treatment (surgery with oncological therapy), whereas 31.9% only received surgery (Table 3). The majority of glioblastoma patients (81.4%) underwent surgery before receiving treatment. Likewise, the majority of patients with medulloblastoma (86.7%), oligodendroglioma grade 3 (92.1%), and astrocytoma grade 3 (88%) also underwent both (surgery and treatment). On the other hand, surgery was the only treatment for the majority of meningioma grade 2 (84.9%) and ependymoma grade 2 (85.3%) cases.

Moreover, we analyzed 918 patients with CNS tumors diagnosed only through radiological imaging, without any histological type or grade. This focus is presented in Supplementary Materials.

## 4. Discussion

This population-based study provides, for the first time in Italy, a comprehensive description of treatment patterns for adults diagnosed with glioblastoma and other central nervous system (CNS) tumors, based on the linkage between the Veneto Cancer Registry (VCR) and administrative healthcare databases. Beyond a descriptive epidemiologic exercise, this integration enabled reconstruction of real-world care pathways and offers a framework for evaluating system-level delivery of neuro-oncology services. By combining data from multiple sources, we were able to reconstruct therapeutic trajectories and provide a multidimensional overview of surgical and oncologic care delivered in routine clinical practice. This work complements our previous population-based analysis on incidence and survival of CNS tumors in the region [5], extending epidemiologic characterization to treatment delivery and management patterns.

Our results confirm that the most frequent malignant CNS tumors in adults are glioblastoma. Among CNS tumors, treatment pathways differ substantially according to histologic type and grade. Most glioblastoma patients underwent surgery followed by combined chemoradiation as adjuvant treatment, in accordance with current international standards [11,12,13]. The proportion of patients undergoing either biopsy or surgery remained relatively stable over time, suggesting homogeneous surgical access within the regional network. However, repeated surgical interventions increased, particularly in glioblastoma. This trend may reflect not only technological advances and perioperative improvements but also evolving clinical decision-making toward aggressive management of recurrence within specialized multidisciplinary settings. These findings are consistent with contemporary real-world evidence supporting surgery at recurrence when feasible.

The proportion of patients receiving both radiotherapy and systemic therapy treatments (about 60%) was lower than typically reported in clinical trials, likely reflecting broader eligibility criteria and comorbidity burden in population cohorts. In addition, although bevacizumab is primarily used in the recurrent glioblastoma setting and represents a recognized therapeutic option, its use appeared limited in this population-based cohort, likely reflecting real-world constraints such as reimbursement policies and patient selection rather than lack of clinical relevance.

The relatively high proportion of untreated patients observed in some tumor subgroups, particularly grade 3 meningiomas and ependymomas, should be interpreted with caution due to small sample sizes. In addition, clinical factors such as age, comorbidities, or poor performance status may have limited the treatment. Notably, in meningiomas, radiotherapy represents the main adjuvant approach, while effective systemic therapies are lacking, which may explain the low use of systemic treatments in this subgroup.

This divergence underscores the importance of registry-based evidence in contextualizing trial-derived expectations and highlights how real-world data can inform realistic benchmarks for healthcare planning and resource allocation. Similar discrepancies between trial and population-based cohorts have been reported across Europe and North America [14,15,16].

For diffuse astrocytomas and oligodendrogliomas, combined treatment predominated and was consistent with guideline-based management [17,18]. A large proportion of low-grade gliomas (grade 2) received postoperative oncologic treatment, reflecting contemporary clinical practice. Importantly, this baseline snapshot provides a reference point against which the impact of emerging targeted therapies can be monitored. Treatment paradigms are expected to evolve following the INDIGO trial results demonstrating benefit from vorasidenib (Servier, Suresnes, France) [19]. Population-based infrastructures such as the present registry linkage will be essential to quantify the adoption of precision therapies and their system-level consequences. Meningiomas were predominantly treated surgically, with adjuvant radiotherapy mainly reserved for higher-grade disease and recurrent lesions. The heterogeneity observed in postoperative radiotherapy use likely reflects institutional practice variation and illustrates how registry data can serve as a tool for identifying areas where guideline dissemination or consensus-building may improve care standardization. Only recently have international and national guidelines, including both European (EANO) and American recommendations, clarified that adjuvant radiotherapy should be reserved for patients with residual disease or incomplete resection in grade 2 tumors, while observation is appropriate after gross total resection. This reflects a therapeutic trend observed in other European registry studies, where postoperative radiotherapy for atypical meningiomas remains heterogeneous and often influenced by surgical extent and institutional policy [20,21,22]. In addition, our data confirm that most patients with medulloblastoma in our cohort underwent postoperative combined therapy (radiotherapy with chemotherapy), reflecting adherence to international standards of care for these rare adult cases [23,24].

Patients diagnosed radiologically without histological confirmation represented more than one-third of cases and were predominantly elderly and untreated. These findings are consistent with prior population studies showing reduced treatment intensity with increasing age and comorbidity [25,26]. From a health-system perspective, this subgroup represents an important indicator population for evaluating referral dynamics, diagnostic accessibility, and equity of treatment access. While treatment decisions should be based on biological rather than chronological age, the absence of histologic verification may limit access to targeted therapies and clinical trials.

Our findings should be interpreted in conjunction with our previous population-based analysis of incidence and survival in the Veneto region [5]. In that study, glioblastoma showed a 5-year relative survival of approximately 5.7%, consistent with the 5–7% reported in the literature, reflecting its well-known aggressive biological behavior. In the present study, only about 60% of glioblastoma patients received combined chemoradiotherapy in routine clinical practice, highlighting variability in real-world treatment delivery. While survival outcomes across CNS tumors are primarily driven by intrinsic tumor biology, these findings suggest that patterns of care observed in the current analysis may have contributed, at least in part, to the population-level outcomes previously reported. Although a formal correlation analysis was beyond the scope of this study, integrating treatment and survival data provides a more comprehensive interpretation of real-world neuro-oncology practice. The integration of cancer registry data with administrative databases allowed mapping of the patient journey and provides an evidence-based framework for healthcare planning aligned with recently adopted regional clinical pathways [27]. Such data infrastructures enable monitoring of treatment variability, benchmarking across regions, and identification of potential access gaps, positioning population-based registries as core components of learning healthcare systems in neuro-oncology. Several limitations should be acknowledged. Molecular data were unavailable, precluding stratified analyses according to genomic subgroups, and administrative data lacked clinical granularity, such as performance status, extent of resection, or clinical outcomes. Nevertheless, the linkage between registry and healthcare databases provides a robust, population-level picture of treatment delivery, reducing selection bias and ensuring near-complete coverage of the regional population. Importantly, these limitations highlight priority areas for future registry evolution rather than diminishing the value of the current analysis.

Future integration of molecular and outcome data will enhance registry utility for monitoring precision oncology implementation and evaluating the effectiveness of novel therapies such as IDH inhibitors (e.g., vorasidenib) in low-grade gliomas [19]. Such expansion would allow transition from descriptive surveillance to actionable quality-of-care analytics and outcome benchmarking.

In conclusion, this study provides a comprehensive population-level overview of real-world CNS tumor management, focusing on glioblastoma. The integration of registry and administrative data offers valuable insights into clinical practice variability, identifies system-level gaps, and supports evidence-based planning of neuro-oncology services. This approach illustrates how population data infrastructures can move beyond surveillance toward active optimization of multidisciplinary care delivery in the era of personalized medicine.

## Figures and Tables

**Table 1:** Characteristics of CNS tumors, by cancer type, sex, and age group.

Cancer Type	Sex		Age Group			Total *N* (%)
	Male *N* (%)	Female *N* (%)	18–49 y *N* (%)	50–69 y *N* (%)	70+ y *N* (%)	
Glioblastoma IDH-wildtype and IDH-mutant	648 (61.4)	407 (38.6)	121 (11.5)	596 (56.5)	338 (32.0)	1055 (64.6)
Astrocytoma grade 2–3	88 (57.5)	65 (42.5)	70 (45.8)	58 (37.9)	25 (16.3)	153 (9.4)
Meningioma grade 2–3	125 (42.0)	173 (58.0)	47 (15.8)	120 (40.2)	131 (44.0)	298 (18.2)
Oligodendroglioma grade 2–3	46 (62.2)	28 (37.8)	38 (51.4)	28 (37.8)	8 (10.8)	74 (4.5)
Ependymoma grade 2–3	23 (59.0)	16 (41.0)	20 (51.3)	17 (43.6)	2 (5.1)	39 (2.4)
CNS embryonal tumor (medulloblastoma)	8 (53.3)	7 (46.7)	11 (73.3)	1 (6.7)	3 (20.0)	15 (0.9)
Total	938 (57.4)	696 (42.6)	307 (18.8)	820 (50.2)	507 (31.0)	1634 (100.0)

**Table 2:** Characteristics of patients by cancer type and oncological treatments (systemic therapy and radiotherapy).

Cancer Type	Only Systemic Therapy *N* (%)	Only Radio *N* (%)	Both *N* (%)	None *N* (%)
Glioblastoma IDH-wildtype and IDH-mutant	149 (14.1)	75 (7.1)	635 (60.2)	196 (18.6)
Astrocytoma grade 2	13 (21.3)	7 (11.5)	22 (36.1)	19 (31.1)
Astrocytoma grade 3	16 (17.4)	5 (5.4)	60 (65.2)	11 (12.0)
Meningioma grade 2	5 (1.8)	33 (12.1)	3 (1.1)	231 (84.9)
Meningioma grade 3	0 (0.0)	11 (42.3)	0 (0.0)	15 (57.7)
Oligodendroglioma grade 2	12 (33.3)	2 (5.6)	9 (25.0)	13 (36.1)
Oligodendroglioma grade 3	12 (31.6)	1 (2.6)	22 (57.9)	3 (7.9)
Ependymoma grade 2	1 (2.9)	2 (5.9)	2 (5.9)	29 (85.3)
Ependymoma grade 3	1 (20.0)	0 (0.0)	2 (40.0)	2 (40.0)
CNS embryonal tumor (medulloblastoma)	2 (13.3)	4 (26.7)	7 (46.7)	2 (13.3)
Total	211 (12.9)	140 (8.6)	762 (46.6)	521 (31.9)

**Table 3:** Characteristics of patients by cancer type, surgical procedure, and oncological treatment (chemotherapy and radiotherapy).

Cancer Type	Only Surgery *N* (%)	Surgery + Treatment *N* (%)
Glioblastoma IDH-wildtype and IDH-mutant	196 (18.6)	859 (81.4)
Astrocytoma grade 2	19 (31.1)	42 (68.9)
Astrocytoma grade 3	11 (12.0)	81 (88.0)
Meningioma grade 2	231 (84.9)	41 (15.1)
Meningioma grade 3	15 (57.7)	11 (42.3)
Oligodendroglioma grade 2	13 (36.1)	23 (63.9)
Oligodendroglioma grade 3	3 (7.9)	35 (92.1)
Ependymoma grade 2	29 (85.3)	5 (14.7)
Ependymoma grade 3	2 (40.0)	3 (60.0)
CNS embryonal tumor (medulloblastoma)	2 (13.3)	13 (86.7)
Total	521 (31.9)	1113 (68.1)

## Data Availability

The datasets generated and/or analyzed during the current study are not publicly available because of privacy reasons. The data presented in this study are available on request from the corresponding author.

## Supplementary Materials

The following supporting information can be downloaded at https://www.mdpi.com/article/10.3390/curroncol33040236/s1, Supplementary Material S1: List of ICD-O-3, ICD-9-CM, and ATC codes used for data extraction; Supplementary Material S2: Patterns of neurosurgical management of patients diagnosed with CNS tumors in 2016-2020. Includes: Table S1. Characteristics of patients by surgical procedure and year of diagnosis; Table S2. Characteristics of patients by cancer type, surgical procedure, and year of diagnosis.; Supplementary Material S3: Focus on patients without microscopic confirmation. Includes Table S3: Characteristics of patients without microscopic confirmation by gender, age group, and oncological treatments (systemic therapy and radiotherapy).

## Abbreviations

The following abbreviations are used in this manuscript:

CNS	Central Nervous System
IARC	International Agency for Research on Cancer
EU	European Union
WHO	World Health Organization
IDH	Isocitrate Dehydrogenase
HA	Hospital Admission
OPS	Outpatient Services
DP	Drug Prescription
HD	Hospital Drugs
ICD-9-CM	International Classification of Diseases, 9th Revision, Clinical Modification
ATC	Anatomical Therapeutic Chemical
VCR	Veneto Cancer Registry
EANO	European Association of Neuro-Oncology
