# Supplementary material for: Real-World Treatment Pathways of Adult Patients with Glioblastoma and Other CNS Tumors: A Population-Based Registry Study
Source: Curr Oncol. 2026 Apr 21;33(4):236. doi: 10.3390/curroncol33040236 (PMC13114890; doi:10.3390/curroncol33040236)
Supplement: Supplementary file 1 [file curroncol-33-00236-s001.zip › curroncol-4226018-supplementary/Supplementary_material_1.pdf]

Supplementary Material S1. List of codes used for data extraction

Coding of the six CNS tumor groups

According to the International Classification of Diseases for Oncology, Third Edition (ICD-O-3) and WHO 2016 classification, the six CNS tumor groups were defined as follows:

- Glioblastoma, IDH-wildtype and IDH-mutant: high-grade gliomas classified under ICD-O-3 morphology codes 9440/3, 9441/3, 9442/3, 9380/3 (grade 4), 9400/3 (grade 4), and 9401/3 (grade 4). These tumors represent the most aggressive form of CNS malignancies.
- Astrocytoma grade 2-3: lower-grade diffuse astrocytic tumors with ICD-O-3 codes 9400/3, 9401/3, and 9411/3, categorized as WHO grade 2 or 3.
- Meningioma grade 2-3: atypical and anaplastic meningiomas, as well as certain borderline or non-malignant variants with more aggressive behavior. The ICD-O-3 codes included were 9530/3, 9530/0-1, 9531/0, 9532/0, 9537/0, 9538/1-3, and 9539/1.
- Oligodendroglioma grade 2-3: tumors of oligodendroglial origin, selected using ICD-O-3 codes 9450/3 and 9451/3, categorized as WHO grade 2 or 3.
- Ependymoma grade 2-3: classic and anaplastic ependymomas identified with codes 9391/3 and 9392/3, categorized as WHO grade 2 or 3.
- CNS embryonal tumor (medulloblastoma): embryonal tumors of the CNS were included if coded as 9470/3, 9471/3, or 9473/3, representing medulloblastoma and its molecular subtypes.

Coding of the surgical procedures

According to the International Classification of Diseases, 9th Revision, Clinical Modification (ICD-9-CM), the surgical procedures were identified from the Hospital Admissions (HA), both in the primary procedure and in the secondary ones, as follows:

- 01.11: Closed [percutaneous] [needle] biopsy of cerebral meninges
- 01.13&01.18: Closed [percutaneous] [needle] biopsy of brain & Other diagnostic procedures on brain and cerebral meninges
- 01.14: Open biopsy of brain
- 01.19: Other diagnostic procedures on skull
- 01.23: Reopening of craniotomy site
- 01.24: Other craniotomy

- 01.25: Other craniectomy
- 01.31: Incision of cerebral meninges
- 01.39: Other incision of brain
- 01.51: Excision of lesion or tissue of cerebral meninges
- 01.52: Hemispherectomy
- 01.53: Lobectomy of brain
- 01.59: Other excision or destruction of lesion or tissue of brain
- 02.99: Other operations on skull, brain, and cerebral meninges
- 03.09: Other exploration and decompression of spinal canal
- 03.4: Excision or destruction of lesion of spinal cord or spinal meninges

Coding of the systemic therapy and radiotherapy

According to the International Classification of Diseases, 9th Revision, Clinical Modification (ICD-9-CM), the systemic therapy and radiotherapy were identified from the Outpatients Services (OPS), as follows:

- 99.25.1, 99.25.2: systemic therapy
- 92.2*: radiotherapy

According to the International Classification of Diseases, 9th Revision, Clinical Modification (ICD-9-CM), the systemic therapy and radiotherapy were, also, identified from the Hospital Admissions (HA), both in the primary diagnoses/procedures and in the secondary ones, as follows:

- Diagnosis codes V58.11-V58.12 or procedure code 99.25: systemic therapy
- diagnosis code V58.0 or procedure codes 92.20-92.29: radiotherapy

Coding of the systemic therapy treatments

According to the International Anatomical Therapeutic Chemical (ATC), the systemic therapy treatments were identified from the Drug Prescriptions (DP) and Hospital Drugs (HD) data flows, as follows:

- L01AX03: Temozolomide
- L01EX05: Regorafenib
- L01AD02: Lomustina
- L01AD05: Fotemustina
