# Supplementary material for: Real-World Treatment Pathways of Adult Patients with Glioblastoma and Other CNS Tumors: A Population-Based Registry Study
Source: Curr Oncol. 2026 Apr 21;33(4):236. doi: 10.3390/curroncol33040236 (PMC13114890; doi:10.3390/curroncol33040236)
Supplement: Supplementary file 1 [file curroncol-33-00236-s001.zip › curroncol-4226018-supplementary/Supplementary_material_2.pdf]

Supplementary Material S2. Patterns of neurosurgical management of patients diagnosed with CNS tumors in 2016-2020

Table S1. Characteristics of patients by surgical procedure (biopsy or surgery) and year of diagnosis.

Year	Only biopsy N (%)	Only 1 surgery N (%)	More than 1 surgery* N (%)	None** N (%)	Total N (%)
**2016**	24 (14.6)	236 (17.9)	13 (8.5)	214 (23.3)	487 (19.1)
**2017**	22 (13.3)	261 (19.8)	16 (10.5)	166 (18.1)	465 (18.2)
**2018**	50 (30.3)	260 (19.7)	41 (27.0)	182 (19.8)	533 (20.9)
**2019**	36 (21.8)	269 (20.4)	39 (25.7)	168 (18.3)	512 (20.1)
**2020**	33 (20.0)	291 (22.1)	43 (28.3)	188 (20.5)	555 (21.7)
**Total**	**165 (6.5)**	**1,317 (51.6)**	**152 (5.9)**	**918 (36.0)**	**2,552**

* The category "**More than 1 surgery**" includes patients who underwent two or more re-operations at different time points. In general, patients who had surgery within three months of the initial biopsy were classified under the "**Only 1 surgery**" group; otherwise, they were included in the "**More than 1 surgery**" category.

** Patients with CNS tumors diagnosed only through radiological imaging, without any histological type and grade

Table S2. Characteristics of patients by cancer type, surgical procedure (biopsy or surgery) and year of diagnosis.

Cancer type	Only biopsy N (%)					Only 1 surgery N (%)					More than 1 surgery* N (%)				
	**2016**	**2017**	**2018**	**2019**	**2020**	**2016**	**2017**	**2018**	**2019**	**2020**	**2016**	**2017**	**2018**	**2019**	**2020**
Glioblastoma IDH-wildtype and IDH-mutant	16 (13.2)	16 (13.2)	39 (32.2)	23 (19.0)	27 (22.3)	148 (17.4)	169 (19.9)	176 (20.7)	166 (19.6)	190 (22.4)	7 (8.2)	9 (10.6)	15 (17.7)	24 (28.2)	30 (35.3)
Astrocytoma grade 2-3	6 (14.3)	6 (14.3)	11 (26.2)	13 (30.9)	6 (14.3)	12 (13.3)	23 (25.6)	16 (17.8)	17 (18.9)	22 (24.4)	4 (19.0)	1 (4.8)	7 (33.3)	6 (28.6)	3 (14.3)
Meningioma grade 2-3	0 (0.0)	0 (0.0)	0 (0.0)	0 (0.0)	0 (0.0)	41 (15.6)	46 (17.6)	49 (18.7)	66 (25.2)	60 (22.9)	1 (2.8)	6 (16.7)	14 (38.9)	8 (22.2)	7 (19.4)
Oligodendroglioma grade 2-3	2 (100.0)	0 (0.0)	0 (0.0)	0 (0.0)	0 (0.0)	19 (29.7)	11 (17.2)	9 (14.1)	14 (21.9)	11 (17.2)	0 (0.0)	0 (0.0)	5 (62.5)	1 (12.5)	2 (25.0)
Ependymoma grade 2-3	0 (0.0)	0 (0.0)	0 (0.0)	0 (0.0)	0 (0.0)	9 (24.3)	9 (24.3)	7 (18.9)	4 (10.8)	8 (21.6)	1 (50.0)	0 (0.0)	0 (0.0)	0 (0.0)	1 (50.0)
CNS embryonal tumor (medulloblastoma)	0 (0.0)	0 (0.0)	0 (0.0)	0 (0.0)	0 (0.0)	7 (46.7)	3 (20.0)	3 (20.0)	2 (13.3)	0 (0.0)	0 (0.0)	0 (0.0)	0 (0.0)	0 (0.0)	0 (0.0)

* The category "**More than 1 surgery**" includes patients who underwent two or more re-operations at different time points. In general, patients who had surgery within three months of the initial biopsy were classified under the "**Only 1 surgery**" group; otherwise, they were included in the "**More than 1 surgery**" category.
