# Supplementary material for: Real-World Treatment Pathways of Adult Patients with Glioblastoma and Other CNS Tumors: A Population-Based Registry Study
Source: Curr Oncol. 2026 Apr 21;33(4):236. doi: 10.3390/curroncol33040236 (PMC13114890; doi:10.3390/curroncol33040236)
Supplement: Supplementary file 1 [file curroncol-33-00236-s001.zip › curroncol-4226018-supplementary/Supplementary_material_3.pdf]

Supplementary Material S3. Focus on patients without microscopic confirmation

We recorded 918 patients with CNS tumors diagnosed only through radiological imaging, without any histological type and grade.

Most of these CNS tumors occurred in older adults, with 702 cases (76.5%) involving people 70 years of age or older (Table S3, mainly men (54.9%). In the first year after diagnosis, the majority (67.5%) of the CNS tumor cases without microscopic confirmation did not undergo oncologic treatment. Most of these patients were older (84.7%). Among those with oncological treatment (n = 298), radiotherapy was administered more frequently than systemic therapy, regardless of age.

Table S3. Characteristics of patients without microscopic confirmation by gender, age group and oncological treatments (systemic therapy and radiotherapy)

	**Only systemic therapy N (%)**	**Only radio N (%)**	**Both N (%)**	**None N (%)**	**Total N (%)**
Male	66 (13.1)	105 (20.8)	14 (2.8)	319 (63.3)	504 (54.9)
Female	41 (9.9)	65 (15.7)	7 (1.7)	301 (72.7)	414 (45.1)
**Total**	**107 (11.7)**	**170 (18.5)**	**21 (2.3)**	**620 (67.5)**	**918 (100.0)**
18-49 y	8 (25.0)	11 (34.4)	3 (9.4)	10 (31.2)	32 (3.5)
50-69 y	21 (11.4)	70 (38.0)	8 (4.4)	85 (46.2)	184 (20.0)
70+ y	78 (11.1)	89 (12.7)	10 (1.4)	525 (74.8)	702 (76.5)
**Total**	**107 (11.7)**	**170 (18.5)**	**21 (2.3)**	**620 (67.5)**	**918 (100.0)**

Men were slightly more likely than women to undergo therapy of any kind (36.7% vs. 27.3%, respectively), while the remaining 72.7% of women and 63.3% of men did not receive any treatment (Table S3). Oncologic treatment was not received by 74.8% of patients aged 70 years and older, compared to 46.2% of patients aged 50-69 and 31.2% of patients aged 18-49. Patients aged 50-69 years had a higher probability of receiving active treatment, especially radiotherapy (38.0%).
